# Decoding the dead: new combined application of strontium and lead isotope analysis to Romano-British cremations from Colchester (UK)

**DOI:** 10.1098/rsos.260591

**Published:** 2026-11-04

**Authors:** Joanna Moore, Glynn J. C. Davis, Hella Eckardt, Geoff M. Nowell, Janet Montgomery

**Affiliations:** ^1^ Department of Archaeology, Durham University, Durham, UK; ^2^ Department of Earth Sciences, Durham University, Durham, UK; ^3^ York Museums Trust, York, UK; ^4^ University of Reading, Berkshire, UK

**Keywords:** Colchester, cremation, Romano-British, lead isotopes, strontium isotopes, mobility, MC-ICP-MS

## Abstract

This paper presents an innovative application of two distinct isotope systems to paired otic capsule and outer petrous bone samples from cremated remains. This dual analysis approach, applied to 22 human cremation burials from Roman Colchester (UK), demonstrates evidence for origins outside Britain for several individuals, shedding fresh light on an emerging capital city within a new province of the Roman Empire. Results have implications for understanding the population dynamics of large Roman provincial urban centres following the conquest period in the north-western provinces, when cremation is the dominant burial practice. The method also allows a detailed reconstruction of individual mobility at different ages within an individual's life.

## 1. Introduction

Previous isotope studies of Romano-British populations have predominantly focused on middle and later Roman period inhumation burials [1–3], reflecting a broader pattern in which geochemical analyses have been applied almost exclusively to inhumation burials (but see e.g. [4–7]). This bias largely stems from the fact that the cremation process significantly restricts what isotopic analyses can be undertaken. Cremation was practised throughout the Roman period and was the dominant burial rite during the first century [8,9]. This analysis therefore reveals new information about the people living in Britain during a pivotal time in the island's history, when there was significant social, cultural and political change following the Roman conquest [10,11]. As part of the *Decoding the Dead* project (2020–2021) undertaken by Colchester Museums in partnership with the University of Reading [12], this study sought to bridge this gap in our understanding and shed light on just how diverse the origins of people in Roman Colchester were, through the application of combined strontium and lead isotope analyses to Romano-British cremation burials for the first time. The significance of this methodological advancement extends beyond Roman funerary contexts, providing new opportunities to investigate populations represented by cremated remains, a practice widely adopted across different cultural, geographic and chronological contexts.

## 2. Archaeological context

Colchester was a major Iron Age centre, a Roman legionary fortress and then the first capital of the new Roman province of *Britannia* [13,14]. The Iron Age people of Camulodunum (modern-day Colchester) had close trade and other links with the continent, and while conquest and direct military occupation here were relatively short-lived, the region continued to see an influx of traders and administrators throughout the Roman period, with settlement forms, material culture and religious practices all undergoing significant change [10]. The majority of cremation burials studied here were acquired by the museum from antiquarian collectors in the late nineteenth century; some are part of the historic collection formed in the early twentieth century.

Most cremated individuals analysed were buried in the first and second century AD, but three cremation deposits of later date, all interred within unusual pewter ossuaries, have also been included. The *Decoding the Dead* project undertook the first analysis of these deposits by modern osteoarchaeological techniques, revealing new information on age, sex and pathologies as well as examining macroscopic bone colour to establish burning intensities and pyre practices (Carroll in [12]). This also identified that a number of these contained multiple individuals, even if only one petrous bone remained for analysis. A fresh assessment of the burial urns and associated objects highlighted the complex ways in which local and non-local, as well as high- and low-status, identities were expressed. Included in this study are the individual buried within the Colchester Vase (figure 1), depicting a gladiatorial *munus* and animal *venatio* [15], and the high-status individual buried under the ‘Mersea Barrow’ cremation deposit with frankincense [16–18]. Also included are three individuals whose cremated remains were interred in vessels known as *ossuaria,* of which only *ca* 40 are known from Britain [19]. Historically assumed to be made of lead, new pXRF and isotope analysis have concluded that three of the Colchester vessels are made of a deliberate lead-tin pewter alloy (table 1). For reasons of space, we will report here on the isotope analysis only, while a separate paper integrates the isotope results more fully with the archaeological and osteological data [12].

**Figure 1.**
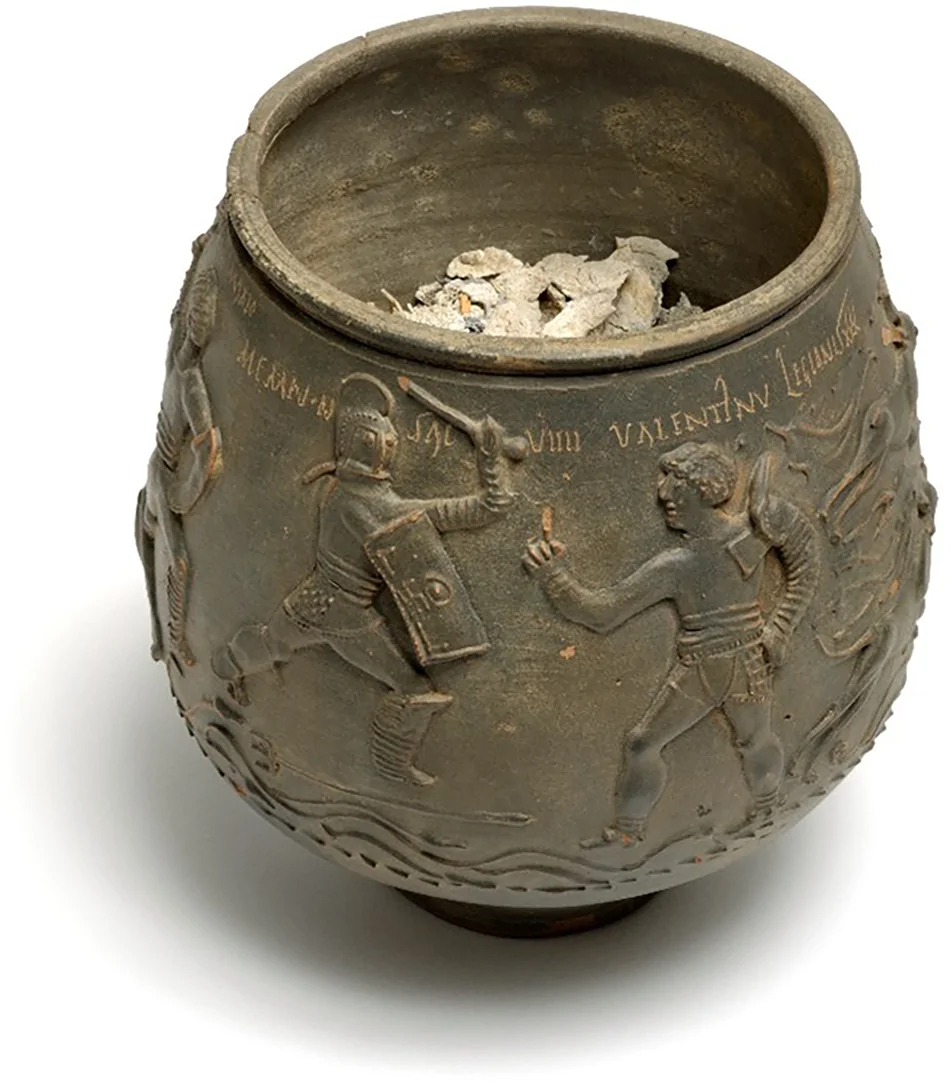
The Colchester Vase depicting a gladiatorial *munus* and animal *venatio*.

**Table 1.** The cremation deposits selected for isotope analysis. Osteological analysis by Carroll (2021); * denotes more than one individual and ? before the sex indicates possible male/female.

AIPRL ID	Sample ID	sex	age	burial vessel	date
Col_1*	1928.363.3	M; U	18+; <13	ceramic face pot	AD 43–80
Col_2*	1930.480.1	F; U	18+; <13	pewter ossuary	AD 200–400
Col_3*	1930.492.1	M; U	18+; <13	pewter ossuary	AD 200–400
Col_4*	2000.72.526.1	U; U	18+; <13	ceramic face pot	AD 43–300
Col_5	34.81.1	F	18+	ceramic pot	AD 125/150–280/320
Col_6	JARMIN.1.1	M	40+	ceramic face pot	AD 43–300
Col_7	JARMIN.3.1	M	18+	ceramic face pot	AD 43–300
Col_8*	JARMIN.89.1	F; M; U	40+; 18+; <13	ceramic pot	AD 43–80
Col_9	JOS.1187.1	U	18+	ceramic pot	AD 43–200/300
Col_10	JOS.32.1	F	40+	ceramic pot	AD 43–69
Col_11	JOS.320.1	U	18+	ceramic pot	AD 117–250/260
Col_12*	JOS.446.1	U; U	40+; 18+	glass bottle	AD 70–150
Col_13	JOS.485.1	M	<13	ceramic pot	AD 54–96
Col_14	JOS.491.1	M	18+	ceramic pot	AD 43–320
Col_15	JOS.781.1	M	18+	ceramic pot	AD 43–120
Col_16	PC.611.1	F	18+	ceramic pot	AD 43–130/140/200
Col_17*	PC.612.1	M; U	18+; <13	ceramic pot	AD 125/150–280/320
Col_18	PC.727.1	M	40+	ceramic pot	AD 110/125–180/210
Col_19	PC.752.1	F	40+	ceramic pot	AD 125/150–280/320
Col_20	1913.2728.1.2	U (?male by [18])	18+ (*ca* 35–45 by [18])	glass jar within lead ossuary	second century (Hadrianic–Antonine)
Col_21	1923.4606.1	U	<13	glass jar with lead lid	AD 100–200
Col_22*	PC.709	M; U	40+; 18+	pewter ossuary	AD 200–400

## 3. Isotope background

### 3.1. Strontium isotopes

The strontium isotope characteristics of plants and local animals in different regions vary depending on the relative contributions of strontium from different underlying rocks and the presence or absence of superficial drift deposits [20]. Strontium is released into the environment through weathering and dissolution processes into the overlying soils and consequently into the food chain (plants, animals and humans). Strontium becomes incorporated into the bioapatite lattice of human bone through ingestion of food and water [21]. Assuming that most of the population's food and drink is sourced locally, the strontium isotope composition in human skeletal material should then reflect the bioavailable strontium isotope ratios in their region of origin [22]. Colchester is located in south-eastern England, in the eastern section of the London Basin (figure 2). This region of England has a varied lithology comprised of Cenozoic sands, gravels and clays known as the London Clay Formation. These types of rocks are expected to produce strontium isotope ratios ranging between 0.7090 and 0.7105 in the plants growing in these geological regions [23]. In the absence of a more detailed, local strontium biosphere map, this range can be considered representative of the local environment. It is therefore expected that people who source most of their food and drink from within this region would have strontium isotope ratios consistent with this range.

**Figure 2.**
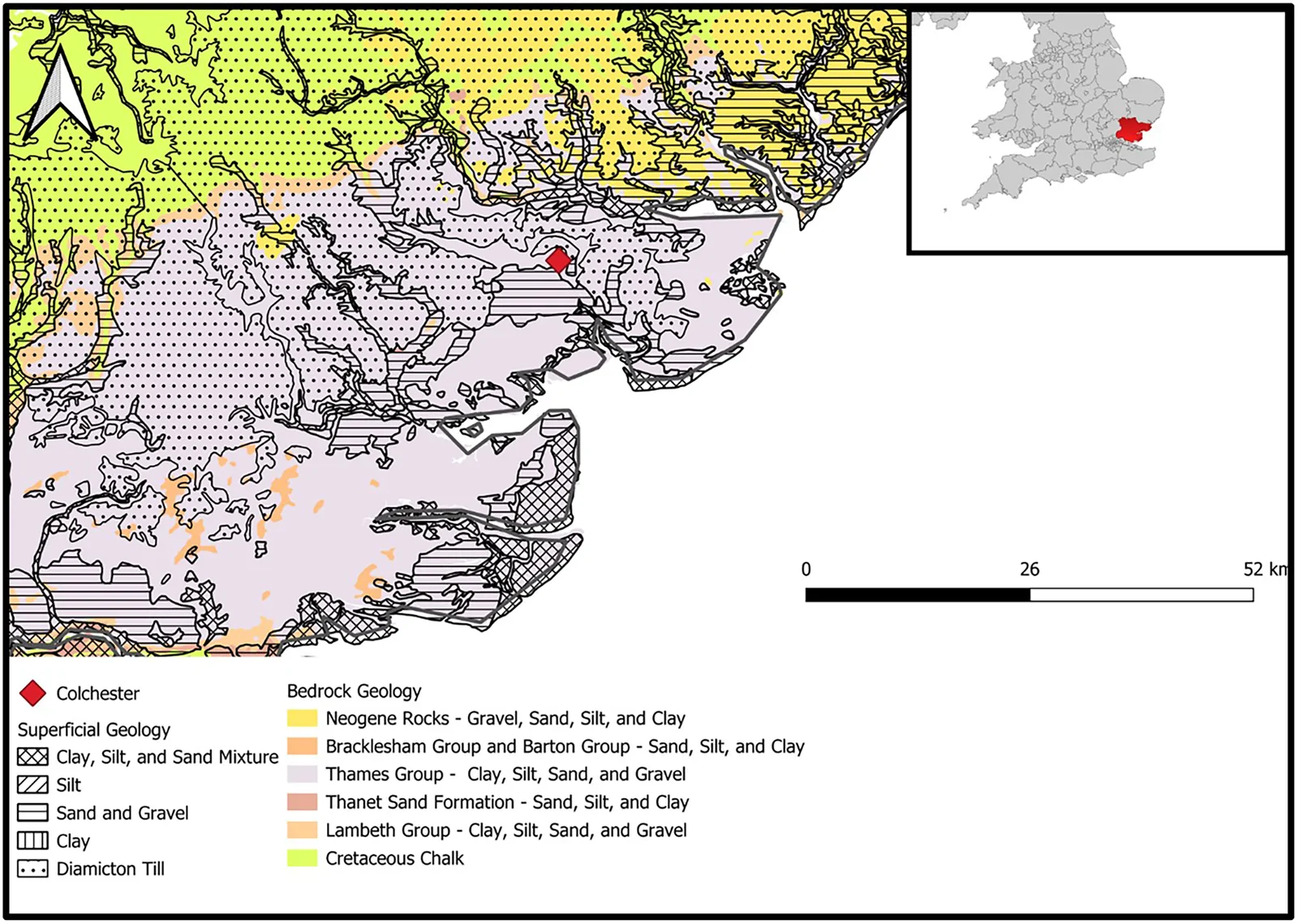
Map of southern Britain showing the geology of Essex and location of Colchester. Based on published maps by BGS with permission from the British Geological Survey.

### 3.2. Lead isotopes

Human lead exposure can be derived from either natural (soil) or anthropogenic (mining, smelting, lead compounds, etc.) sources. Natural exposure tends to be characterized by low lead concentrations, typically below 0.87 ppm and reflects the composition of the local soils [24–26]. Conversely, anthropogenic exposure results in increased concentrations and clustered or ‘culturally focused’ lead isotope ratios that represent an averaging of the pollutant sources that may or may not match those found naturally in the local environment [25–28]. A diachronic study of lead isotope ratios in British archaeological populations has established that anthropogenic ^207^Pb/^206^Pb in individuals exposed to English and Welsh lead ore ranges between 0.845 and 0.849 [25]. While Britain has a rather well-defined human lead isotope range, there are other regions both within and without the Roman Empire that are poorly defined, a limitation that can make determining origins challenging.

## 4. Strontium and lead in cremated bone

Certain intrinsic features of bone, such as porosity, density and surface area, render bone highly reactive with its surroundings, readily exchanging mineral and organic elements with its burial environment [29,30]. For this reason, tooth enamel, with its high crystallinity and subsequent resistance to diagenetic alteration, is the tissue type primarily sampled to address questions about mobility in the past. Although this has limited research to childhood mobility in inhumations, work on cremated remains has demonstrated that fully calcined bone undergoes changes to the crystalline properties of the bioapatite when heated to temperatures of greater than or equal to 600°C [31], making it more resistant to diagenetic alteration in the burial environment than unburned bone [32,33]. Strontium isotope analysis on such samples has demonstrated that calcined bone, once adequately pretreated, can be used to assess the geographic origins of archaeological cremated remains ([7,34–39]). While this development provided a step-change in how isotopic analysis can be applied to cremations, the use of a single isotope system for provenance is limiting, as many places across the world share similar, if not the same, strontium isotope ratios. In this regard, lead isotope analysis may provide an additional dimension to the geochemical provenance of archaeological cremated remains, particularly in Roman period Britain where human lead isotopes have been extensively studied (e.g. [25,26,40–42]). However, there are concerns that the cremation process may alter the *in vivo* lead composition of cremated remains and does not render calcined bone resistant to postmortem uptake of lead from the burial environment in the same way it does for strontium [43]. To date, no papers have been identified that specifically address this in any great detail, but it is known that while metallic lead has a low melting point of 327.5°C, lead is not present in bone in its elemental form [44,45]. Metabolic lead (Pb^2+^) substitutes for calcium (Ca^2+^) in the mineral matrix, forming calcium-lead apatite, a stable insoluble compound with a high melting point over 1100°C [46,47]. Multiple papers have demonstrated that the relatively high temperatures needed to smelt copper or bronze (greater than 950°C) do not significantly fractionate lead isotope ratios, allowing archaeometallurgical provenance studies to be conducted on metal artefacts [48,49]. Still, it remains to be demonstrated that the same can be said for lead in bioapatite, as osteological analyses of cremation deposits and burning experiments have indicated that historic pyres can reach up to 1000°C, with most maintaining temperatures between 700 and 900°C [43,50–52]. For the few studies that have employed lead isotope ratios on cremation deposits, the results are promising [53–55].

The nature of cremated remains not only limits the isotope systems but also the skeletal elements that can be used in their osteological analyses. For instance, teeth are often not available for study, making the assessment of childhood origins difficult. They are either usually destroyed during the cremation process or can be absent owing to their small size and the sequence of the Roman funerary process of cremation ([56,57]; though see [58] and [59]). However, in the absence of teeth, the otic capsule has proven to provide a suitable alternative; this part of the cranium is found within the petrous part of the temporal bone and is often present in the burial. The otic capsule of the petrous bone forms from 16–18 weeks in utero and does not remodel after the first two years of life [60–62]. Therefore, the strontium isotope ratios measured from this part of the skeleton reflect early childhood diet, and partially incorporated strontium from the mother. While the development and turnover of the otic capsule is well defined, less is known about the rest of the petrous bone. Although modern studies have determined that the petrous bone tends to be fully grown by the ages of 12 to 15 years [63], there is no research into its turnover rate or how that might alter throughout life. If, like other dense bones in the body (e.g. femur), the turnover rate is slow, then it is possible that the isotope ratios measured in the outer surface of the petrous reflect a mix of strontium from adolescence to death [64–66]. To this end, movement throughout childhood and later life was investigated using, where possible, paired analysis of strontium isotope ratios from the otic capsule (early childhood) and the outer surface of the petrous portion (adolescence). Lead isotope ratios were also measured in the fully calcined samples taken from the outer surface of the petrous portion.

## 5. Material and Methods

### 5.1. Samples

Petrous bones from 22 Romano-British cremation burials (table 1) were sampled for strontium and lead isotope analysis. Initial analysis was conducted on the otic capsule inside the petrous part. However, when exposed, not all otic capsules were fully calcined, so a second sample was taken from the fully calcined outer surface of the petrous part. Calcination was assessed macroscopically based on bone colour, with fully calcined bone having a completely white and chalky appearance [67]. This paired sampling allowed a unique opportunity to compare fully calcined versus partially burnt bone from the same skeletal element, as well as facilitating the assessment of mobility over two distinct periods of life.

Lead isotope analysis of the base and lid of three lead-rich pewter urns was also conducted to determine the provenance of the lead used in these burial containers and to assess whether there had been contamination through the burial environment.

### 5.2. Sample pretreatment

The sampling of the otic capsule followed the protocol described by Snoeck *et al.* [33] and Veselka *et al.* [68]. The pars petrosa was first cut transversally through the internal auditory meatus with a clean dental saw to access the cross section of the cochlea. The exposed features were carefully cleaned by removing a thin surface layer of bone. Subsequently, bone powder was collected with a clean dental burr from the surface of the internal canals (*ca* 0.5 mm).

The powdered samples from the otic capsule and the outer surface of the petrous part were transferred to clean glass vials and underwent ultrasonication in Milli-Q water for three 10-minute episodes. This was followed by one 10-minute ultrasonication with 0.1 M acetic acid, and a repeat of the three 10-minute Milli-Q runs. In between each run, the samples were rinsed with Milli-Q water. To prevent material loss, the samples were centrifuged before water was discarded. Once completely clean, the bone powder was transferred to clean 1.5 ml sample tubes and freeze-dried overnight.

### 5.3. Analytical procedures

Strontium and lead from pre-leached powdered bone samples were isolated using ion chromatography on columns loaded with Eichrom© resin after dissolution overnight in 3 M HNO_3_ at the Arthur Holmes Isotope Geology Laboratory (AHIGL), Durham University.

Strontium isotope measurements were made on a Thermo Scientific Neptune MC-ICP-MS in static mode with each measurement comprising a single block of 50 cycles. Instrumental mass bias was corrected for using an ^88^Sr/^86^Sr ratio of 8.375209 and an exponential law. Corrections for isobaric interferences from Rb and Kr on ^87^Sr and ^86^Sr were performed using ^85^Rb and ^83^Kr as the monitor masses but were insignificant. In all samples, the ^85^Rb intensity was less than 19.5 mV with an average ^85^Rb/^88^Sr ratio of less than 0.02. ^83^Kr was between 0.08 and 0.13 mV in all samples. Samples were analysed during a single analytical session during which the average ^87^Sr/^86^Sr ratio and reproducibility for the international isotope reference material NBS987 was 0.710258 ± 0.000018 (2*σ*; *n* = 10). Strontium isotope data for samples are normalized to the accepted value for NBS987 of 0.710240.

A purified lead fraction was collected from the same Eichrom resin column used to isolate the strontium fraction. The samples were analysed on a Thermo Scientific Neptune Plus with a high-sensitivity sample introduction system comprising an Aridus II desolvating nebulizer and the high-sensitivity ‘X’ skimmer cone yielding approximately 1200 V total Pb signal per ppm of Pb in the sample. Each measurement comprised a static multi-collection routine, with ^205^Tl in the axial detector, of one block of 50 cycles with an integration time of 4 s per cycle. To correct for instrumental mass bias, samples were spiked with a thallium (Tl) standard to achieve a Pb/Tl ratio of approximately 12. Since the mass bias behaviour of Pb and Tl during MC-ICPMS is very slightly different, it is necessary to optimize the ^205^Tl/^203^Tl ratio in each analytical session to correct for this difference and essentially force the mass bias factor (*β*) for Tl to equal that for Pb (Tl*β*/Pb*β* = 1). This is accomplished by adjusting the ^205^Tl/^203^Tl ratio to minimize the offset between each measured Pb isotope ratio and the accepted Pb isotope ratio for the international isotope reference material NBS981. In our case, we use the Pb isotope ratios of [69] as the accepted values for NBS 981. These optimized ^205^Tl/^203^Tl ratios are then used to correct the mass bias on all unknown sample Pb ratios. The samples were analysed in a single analytical session during which the standard NBS981, diluted to 50 ppb, was used and mean isotope ratios of the standards were recorded. The mean isotope ratios were ^206^Pb/^204^Pb = 16.94161 ± 0.000919, ^207^Pb/^204^Pb = 15.49983 ± 0.001125, ^208^Pb/^204^Pb = 36.72566 ± 0.003525, ^208^Pb/^206^Pb = 0.91490 ± 0.000021 and ^208^Pb/^206^Pb = 2.16779 ± 0.000095 (2*σ*; *n* = 16).

For all samples, major and trace elements were measured using a 50 µl aliquot of the digested sample removed before strontium and lead purification and made up to 1 ml 3 M HNO_3_. An aliquot of each sample was then diluted to give approximately 5 mg per 10 ml (0.5 mg ml^–1^) to keep the total dissolved solids at a sufficiently low level to reduce matrix suppression effects within the analytical instruments. Diluted samples were then analysed via ICP-MS (Thermo Scientific X-series) using an external calibration for the minor elements. The major elements Ca and P were determined via ICP emission spectroscopy (Thermo Scientific iCAP 6000) owing to the higher concentration. Both instruments were optimized prior to use to maximize sensitivity and reduce potential spectroscopic interferences. All measurements were reported in ng mg^–1^, which is equivalent to parts per million (ppm).

## 6. Results and discussion

The outer surface of all cremated petrous bones were fully calcined and are therefore likely to have retained their *in vivo* strontium isotope characteristics [33]. However, the internal structures of the otic capsule, which are better protected from the extreme heat of cremation, were not fully calcined in all samples (supplementary material). Consequently, lead isotope ratios were not measured in the otic capsule samples, and it is acknowledged that the strontium isotope ratios from the otic capsules may have been diagenetically altered, although research has indicated a promising resistance to postmortem change [70]. Incorporation of labile strontium from the burial environment into bone would pull strontium isotope ratios towards the expected local range and may increase strontium concentrations if by addition rather than replacement [22]. However, many of the otic capsule samples exhibit strontium isotope ratios outside the expected range for Colchester, suggesting that they have retained some, if not all, of their *in vivo* characteristics.

### 6.1. Strontium

The Colchester strontium concentrations range from 44 to 192 ppm (median = 89.4 ppm, IQR = 32.0), which is within the range expected for human tooth enamel from archaeological populations in Britain [71]. In most individuals (*n* = 10/14) where otic capsule and outer surface samples were taken, the strontium concentrations were significantly higher (*t* (17) = –2.36, *p* = 0.03) in the outer petrous bone samples than the otic capsule samples (figure 3). Three individuals (COL_6, 13 and 15) have slightly lower outer surface concentrations compared with their otic capsule; however, these values are within analytical uncertainty of each other and therefore could be considered the same. It is well documented that diets high in protein and calcium suppress strontium uptake [72]. Therefore, it is possible that the lower strontium concentrations observed in most of the otic capsules are a result of high milk diets in infancy and early childhood (less than or equal to 2 years).

**Figure 3.**
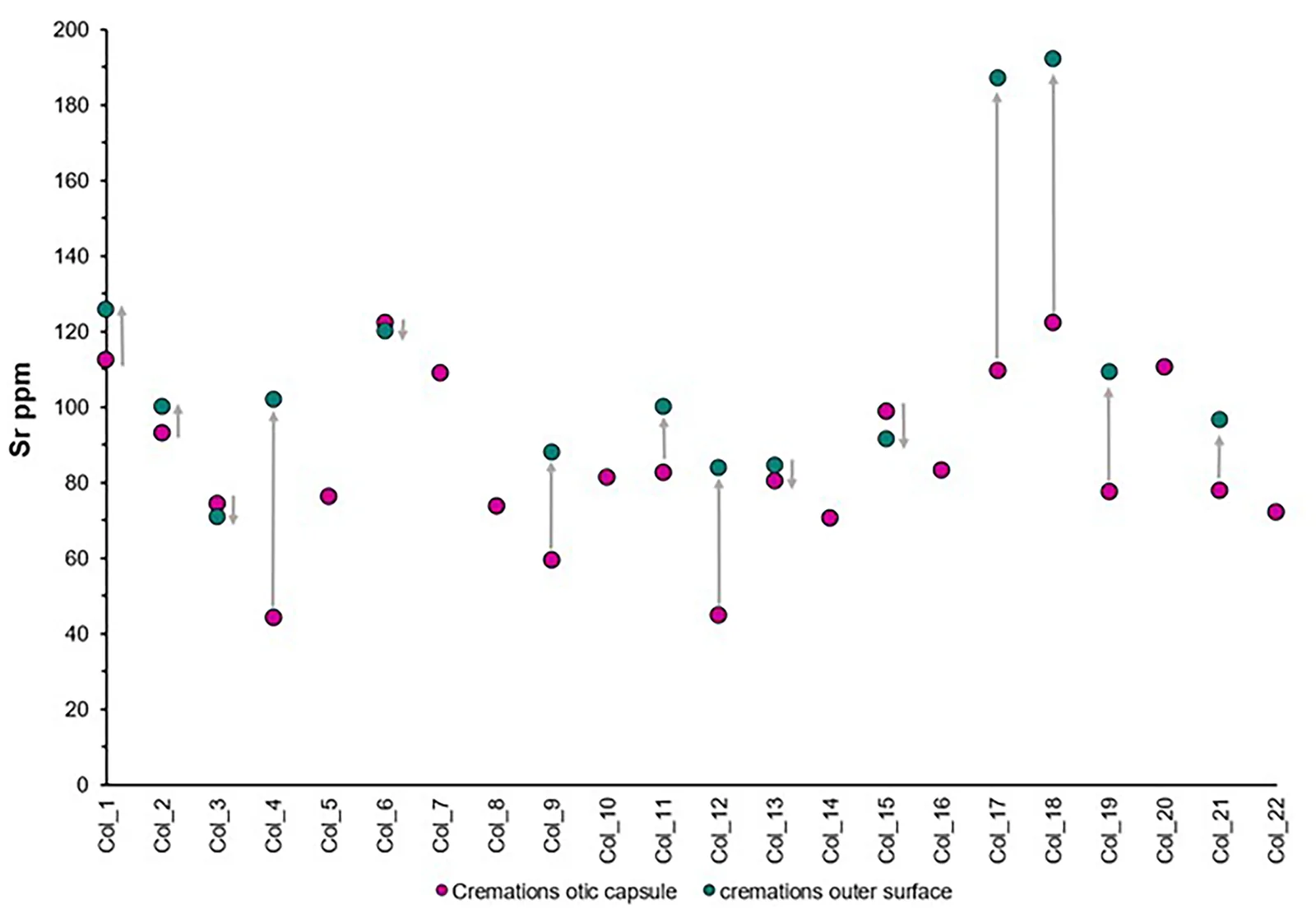
Strontium concentrations (Sr ppm) in the otic capsule and outer surface of the petrous bone from the Colchester cremation deposits. Grey arrows indicate the earliest-forming sample (otic capsule) to the later-forming sample (outer surface).

The strontium isotope ratio data for the Colchester cremated remains range from 0.70848 to 0.71267 (median = 0.70994, IQR = 0.00082), which is within the range expected for humans in Britain [71]. The majority of the individuals have otic capsule and outer petrous bone strontium isotope ratios consistent with a childhood and later life spent in Colchester or southern Essex. These individuals also plot closely with Romano-British individuals recovered in London (figure 4A), where the local geology, like Colchester, is predominantly London Clay. However, it is important to note that there are other places where the strontium isotope ratios seen in these individuals can be found, as they are very common in humans across Europe.

**Figure 4.**
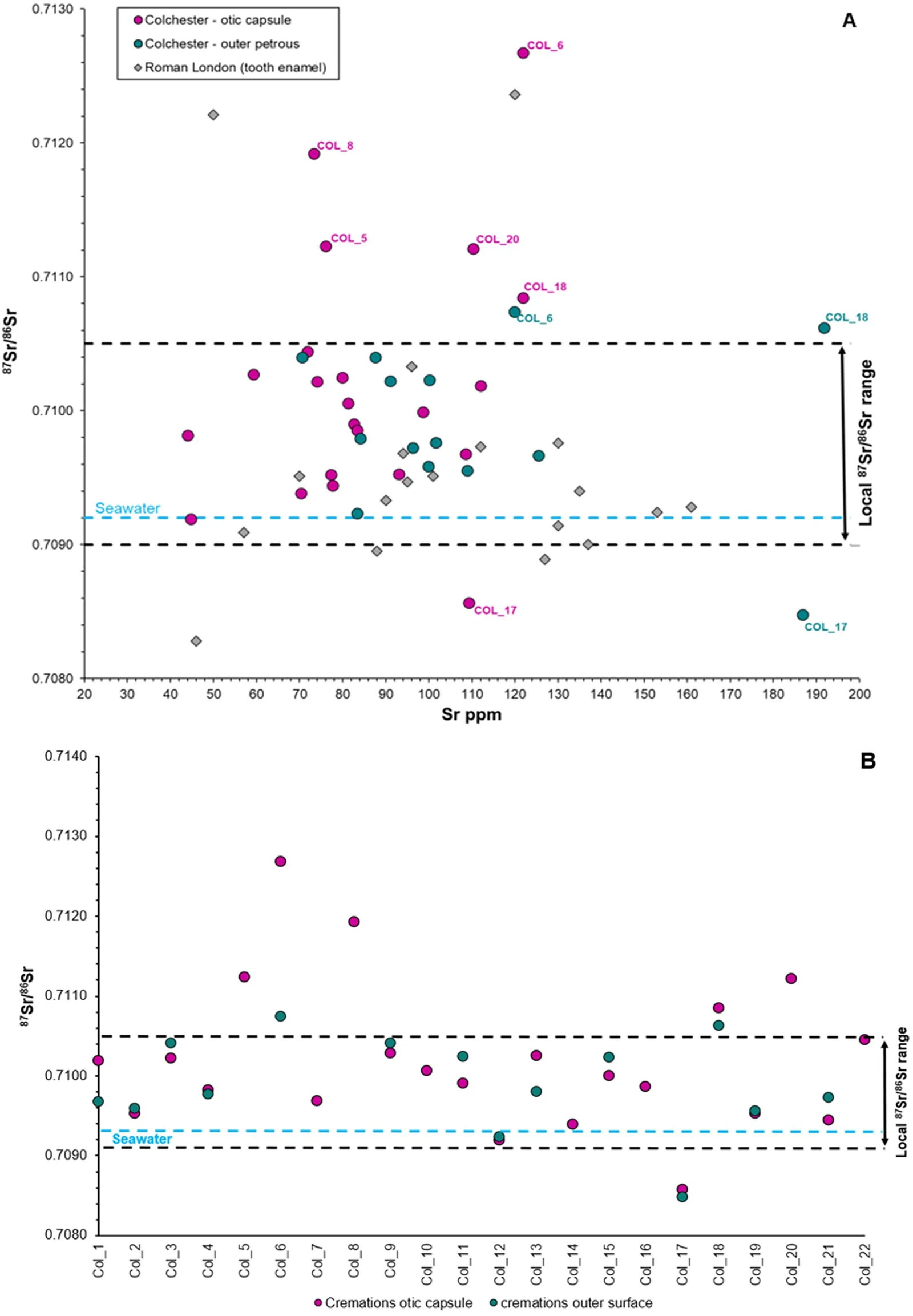
(A) Colchester cremations strontium isotope data alongside the bioavailable strontium isotope range for Colchester [23] and Roman-period inhumation burials from London [42]. The analytical error for ^87^Sr/^86^Sr is within the symbol. (B) Colchester cremation deposit strontium isotope data alongside the bioavailable strontium isotope range for Colchester [23]. The analytical error for ^87^Sr/^86^Sr is within the symbol.

Six individuals have strontium isotope ratios that fall outside the expected local range for Colchester. Individuals COL_5, 6, 8, 18 and 20 have higher strontium isotope ratios indicating origins in regions with older geology such as Wales, Scotland and the south-west of England. In all instances where both an otic capsule and an outer petrous bone sample were available, the otic capsule exhibits more radiogenic values, with a shift towards the local bioavailable range in the petrous bone (figure 4B). As the petrous bone samples were fully calcined, it is unlikely that this shift towards local values is a result of diagenetic alteration. Rather, this shift indicates movement to Colchester or somewhere with a similar geology after the mineralization of the otic capsule (approx. 2 years) and during the growth and remodelling of the petrous bone as it matured to its adult size. One individual (COL_17) has lower strontium isotope ratios than expected for the region in both their otic capsule and outer petrous bone. This indicates childhood origins in an area dominated by rocks of marine origin such as chalk or limestone. Such values could be expected in regions of north Essex, but these lithologies crop out extensively across the south and east of Britain and are widely found around the Mediterranean Sea, e.g. Italy, Greece, Egypt and Lebanon.

### 6.2. Lead

The Colchester cremation samples (excluding five individuals associated with lead and pewter burial vessels, see below), had lead concentrations ranging between 0.4 and 47.1 ppm. Despite the loss of organic material during the cremation process, which will have concentrated the trace elements in the bone mineral, the Colchester samples have lead concentrations consistent with those measured in tooth enamel from contemporaneous individuals from across the Empire [26,41,42,73,74]. Most samples have lead concentrations above 0.87 ppm, indicative of anthropogenic exposure and suggesting residency in a polluted environment [24,26]. However, the otic capsule samples from cremations COL_11, 13, 16 and 19 have extremely low lead concentrations of 0.4 to 0.6 ppm. Colchester was a major urban centre, having been the site of a legionary fortress in the immediate post-conquest period [13,14]. As in many urban centres, people may have been exposed to lead through general metalworking [14,75]. Most relevant artefacts such as pewter vessels and lead coffins post-date the cremations, but Colchester had a pressurized water system consisting of wooden water mains as well as lead pipes and tanks [14,76]. Therefore, such limited exposure to lead in and around Colchester is unexpected. Lead burdens below 0.87 ppm are usually seen in Britain in prehistoric or early medieval populations that correspond with a ‘clean’ period in the atmospheric record where the mining, smelting and use of lead and lead compounds was limited [26]. In Roman individuals, lead concentrations this low are uncommon in urban locations and strongly suggest that these individuals spent their early childhoods in a region with little to no anthropogenic lead pollution (i.e. a rural settlement) or originated from a region outside the Roman Empire. However, it must be noted that a limited number of experimental studies have found that burning at high temperatures (greater than 900°C) and the use of acid pretreatments can reduce trace element concentrations in cremated bone [43]; therefore, caution is needed when interpreting such data.

The four individuals deposited inside the lead and pewter ossuaries (COL_2, COL_3, COL_20 and COL_22) and one individual inside a glass vessel with a lead lid (COL_21) had high lead concentrations ranging between 123 and 3029 ppm. When the lead isotope ratios from the three fully calcined individuals are plotted alongside the data from associated pewter ossuaries (figure 5), the bone and ossuary ratios cluster tightly together where the English and Welsh lead ore fields overlap, and within the culturally focused anthropogenic range expected of archaeological British populations (^207^Pb/^206^Pb = 0.845 to 0.849). This indicates that the pewter ossuaries were made using British lead sources and is a clear indication of postmortem contamination of the human remains from their burial containers that could not be removed with pretreatment. Therefore, these five individuals have been excluded from further analysis regarding childhood mobility based on lead isotopes.

**Figure 5.**
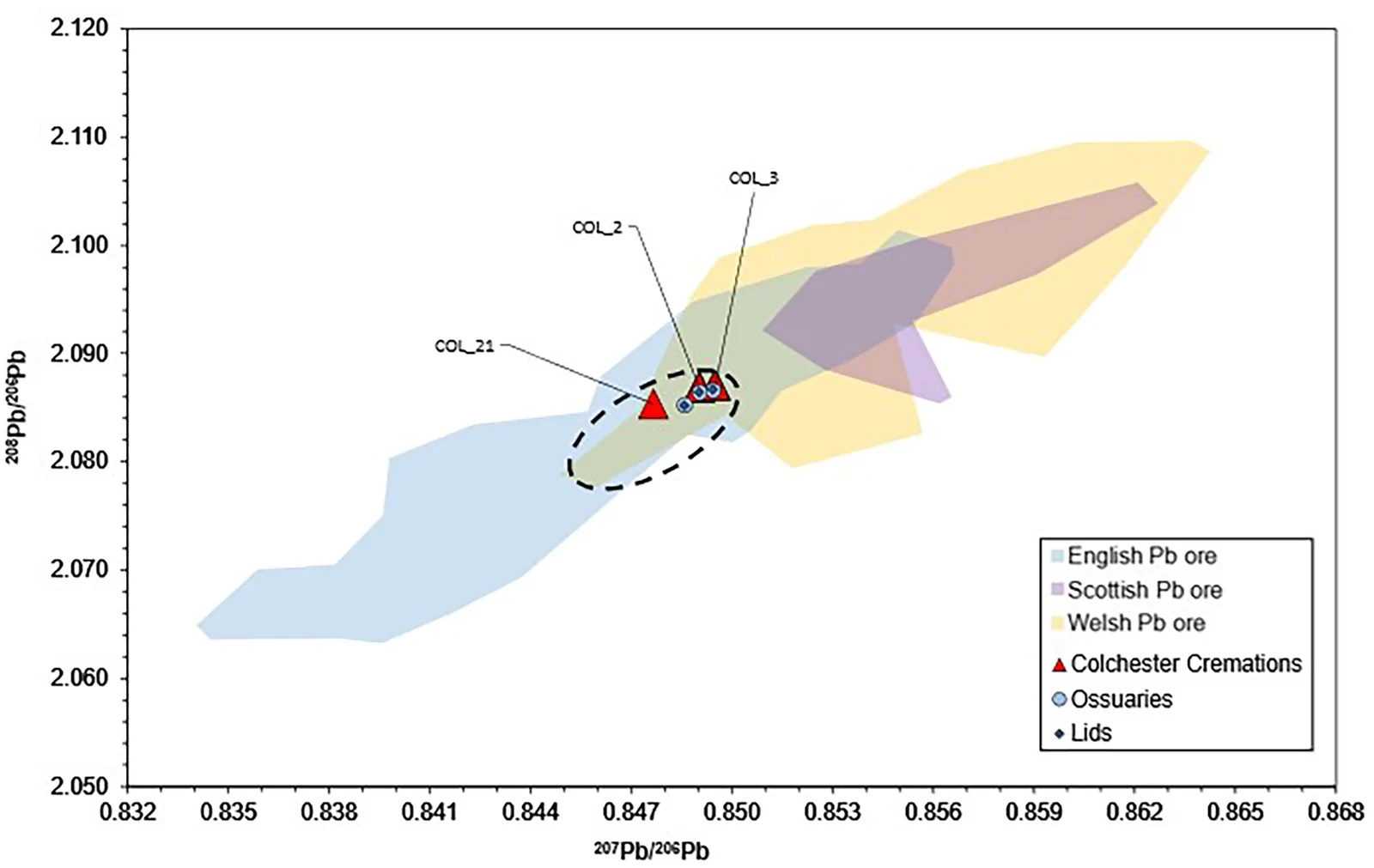
^208^Pb/^206^Pb against ^207^Pb/^206^Pb showing the relationship between the pewter ossuaries and their associated human remains. The shaded areas denote convex hulls representing the lead ore data from Rohl [77], and the dashed ellipse delineates the range in lead isotope ratios expected for Romano-British individuals as defined by Montgomery *et al.* [26].

Lead isotope ratios tend to form clusters or linear arrays that spread over a wide range of values, often resulting in an overlap between lead ore fields from different countries. Owing to this overlap, which is evident in both tooth enamel and lead ore data, lead isotope ratios are not country-specific. Nevertheless, lead isotope ratios can be useful in distinguishing between broad regions of Europe. A comparison of the Colchester cremations' lead isotope ratios in the outer surface of the petrous bone with data from British and circum-Mediterranean lead ores, as well as tooth enamel from Roman period skeletons recovered in western European regions of the Roman Empire, highlights how these Colchester individuals represent a diverse group of people (figure 6). Over half of the Colchester individuals cluster tightly in the centre of the English lead ore field and with the main group of Romano-British tooth enamel samples, suggesting that these individuals were living in England during their adolescence/early adulthood. COL_1 has lower ^206^Pb/^204^Pb than the main cluster of Colchester individuals. The lead and strontium isotope ratios for COL_1 plot closely with Romano-British samples from Musselburgh, Scotland and Dog Hole Cave, Cumbria and could suggest more northerly origins for this individual. Three individuals (COL_12, 18 and 19) have higher ^208^Pb/^204^Pb and ^207^Pb/^204^Pb than those usually seen in British populations. Although these values sit on the edge of the English lead ore field, they overlap with lead isotope ratios of younger lead ores from Mediterranean regions of Europe (figure 6). Lead isotope ratios as high as those measured in COL_12, 18 and 19 are not consistent with origins in Britain and plot more closely with Roman tooth enamel samples from Spanish and Italian populations. This strongly suggests that these individuals originate from outside Britain because while there is evidence for exportation of lead from Britain [83], there is no published evidence for imported lead artefacts in Romano-British contexts (P Bray, personal communication).

**Figure 6.**
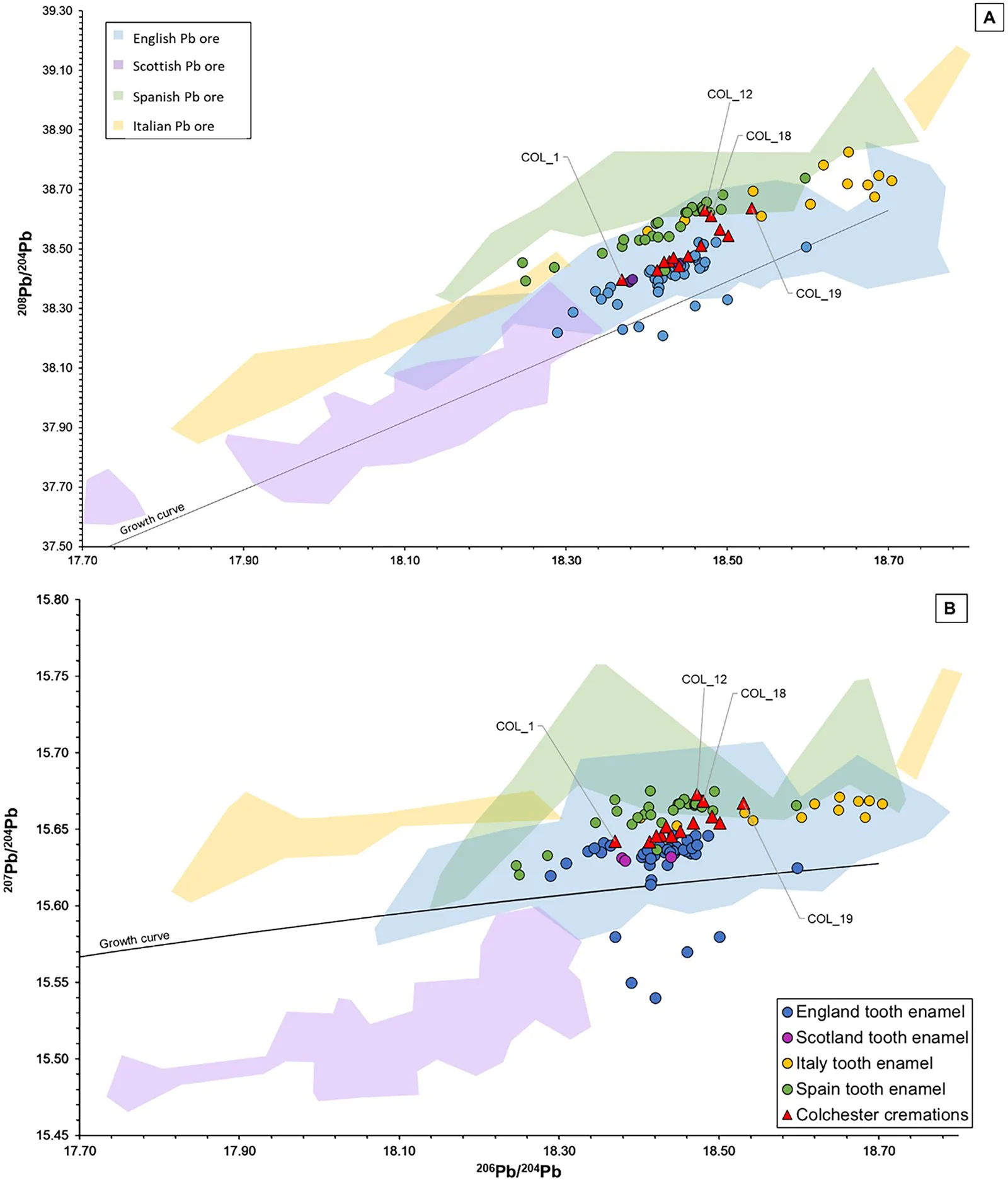
^208^Pb/^204^Pb (A) and ^207^Pb/^204^Pb (B) against ^206^Pb/^204^Pb showing how tooth enamel samples from Britain group with regional lead ore; the [78] growth curve is included for reference. Comparative tooth enamel data from Moore *et al.* [41]; O'Regan *et al.* [74]; Shaw *et al.* [42]; Montgomery *et al.* [26]; Hoare [79]; Moore [40]; and Montgomery [25]. The shaded areas denote convex hulls representing the lead ore data from Rohl [77]; Stos-Gale & Gale [49]; Boni & Koeppel [80]; Montreo-Ruiz *et al*. [81] and Baron *et al.* [82].

General trends in European tooth enamel lead isotope ratios have been shown to relate to major orogenic events, during which lead ores are often formed [27,40,84]. Individuals from regions with older, Hercynian, Cambrian or Precambrian orogeny (e.g. Britain, France, Germany and Scandinavia) tend to have lower ^208^Pb/^206^Pb ratios than individuals from regions with younger, Alpine orogeny (e.g. Spain, Italy and Romania) [40,85]. This is clearly demonstrated in figure 7 where Roman tooth enamel samples from Italy and Spain are plotted in a linear array above contemporaneous samples from England, Scotland and France. Plotting the Colchester cremations alongside this data gives a clearer visual representation of how different the four main outliers are from the main cluster of Colchester individuals. Most of the Colchester individuals group tightly within the constrained English/Welsh anthropogenic range identified by Montgomery *et al.* [26], suggesting that these individuals grew up in either England or Wales. COL_1 has a higher ^207^Pb/^206^Pb ratio, plotting outside this anthropogenic range but is still consistent with growing up in an older orogenic zone (e.g. northern Britain or France). COL_12, 18 and 19 separate much more clearly in figure 7, with higher ^208^Pb/^206^Pb ratios than other Romano-British individuals. These three individuals most likely have childhood origins in the Mediterranean regions of the Roman Empire, with similar values seen in tooth enamel from contemporaneous individuals from Spain, Italy and Lebanon [40].

**Figure 7.**
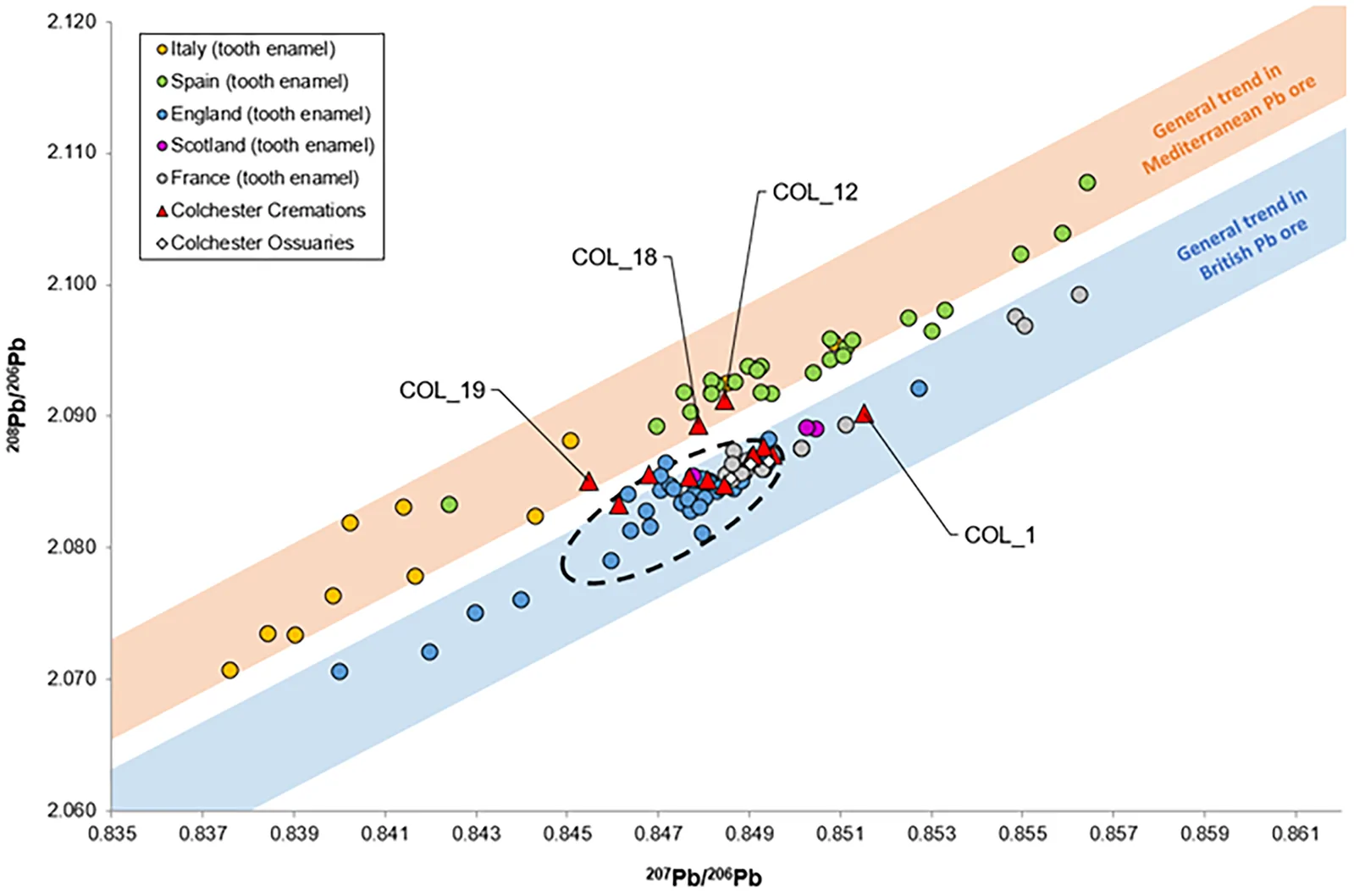
^208^Pb/^206^Pb versus ^207^Pb/^206^Pb showing the relationship between Roman human tooth enamel samples and European lead ore fields. The dashed ellipse delineates the range in lead isotope ratios expected for Romano-British individuals as defined by Montgomery *et al.* [26]. Comparative Roman tooth enamel data taken from Moore [40]; Moore *et al.* [41]; O'Regan *et al.* [74]; Shaw *et al.* [42] and Montgomery *et al.* ([26], [86]). The shaded bands represent observed patterns in lead ore data taken from Rohl [77] and Stos-Gale & Gale [49].

It is important to note that the different sources of evidence do not coalesce consistently to identify incomers. This is in part owing to the fact that lead and strontium isotopes provide different information regarding an individual's life course. Strontium represents the geology of the land from which an individual's food and water were sourced, while lead tends to represent the dominant ore sources exploited by a particular population. This study has shown that by using a combined isotope approach it is possible to improve not only our ability to identify migrants within cremated assemblages but also narrow down possible regions of origin for these individuals. Out of the six individuals identified as non-local on the basis of strontium alone, only one (COL_18, the individual associated with the Colchester Vase) has both high strontium and lead isotope ratios that suggest an Italian or Spanish origin for this individual. The remaining five outliers (COL_5, 6, 8, 17 and 20) are clearly not local to Colchester but have lead isotope ratios indicating that they likely originate from somewhere else in Britain. Conversely, COL_1, 12 and 19 have lead isotope ratios that suggest origins outside Britain but display strontium isotope ratios consistent with Colchester. These non-local lead isotope ratios coupled with apparently local strontium isotope ratios most likely reflect movement from a region outside Britain that has a similar underlying geology to that found in Colchester. This is because strontium isotope ratios primarily reflect the age and composition of local bedrock; therefore, strontium isotope ratios indistinguishable from those expected locally can occur across geographically distant regions. Additionally, COL_19 also exhibits a low lead concentration of 0.6 ppm in their otic capsule. Lead concentrations below 0.87 ppm are uncommon in Roman contexts and suggest that this individual spent their early childhood (less than or equal to 2 years) in a rural location; this may have been in a Mediterranean region of the Empire judging from the lead isotope ratio, although one would assume significant use of, and exposure to, lead within most of the Empire. In some cases, the archaeological evidence agrees with the isotope data (e.g. COL_20, the richly furnished ‘Mersea Barrow’ burial, containing frankincense, has a high strontium isotope ratio) but in others the grave goods appear to be local even where strontium isotope ratios suggest immigration (COL_5). A full examination of these complex patterns is beyond the scope of this paper but will be addressed in a forthcoming paper that combines artefactual and epigraphic data as well as considering the age, sex and pathologies of these individuals [12].

## 7. Conclusions

Colchester was a major military and urban centre, with epigraphic, historical and artefactual evidence for incomers from many parts of the Empire. However, with the conventional use of just strontium isotope analysis of cremated remains, it is often difficult to narrow down possible places of origin outside Britain, especially in Roman cities where populations are considered to have been highly diverse and where the geology is not overly distinctive [1,87]. This study presents the innovative application of two distinct isotope systems to paired otic capsule and outer petrous bone samples from cremated remains, allowing for the first time a detailed reconstruction of individual mobility at different ages within an individual's life. By moving beyond the conventional use of just strontium isotopes, it enables unprecedented resolution in identifying movement from cremated remains. In doing so, this approach marks a major methodological advancement, pushing the boundaries of how past mobility is understood and reconstructed in populations where cremation is the dominant burial rite. Here, four migrants to Britain were identified on the basis of their lead isotope characteristics (COL_1, 12, 18 and 19), which would have otherwise gone undetected, and a further three potentially from outside the Empire on the basis of very low lead concentrations (COL_11, 13 and 16). Alternatively, it is possible that those with low lead concentrations grew up in a rural location where lives were less influenced by the urban and industrial developments and cultural artefacts associated with Roman Britain. While both strontium and lead data only point to non-local origin in one case (the Colchester Vase burial COL_ 18), the overall patterning substantiated the supposed diverse nature of Colchester's population during the Roman period. The identification of several individuals with lead isotope ratios outside the expected range for anthropogenic exposure to British lead sources is a strong indication that fully calcined bone retains its *in vivo* lead characteristics and can be used to trace mobility in cremated remains. Although demonstrated here in Romano-British contexts, this methodology has the potential to be globally and temporally transferable, providing a powerful new tool with the potential to reshape mobility research across archaeology and related disciplines. It has particular potential for the Roman world, where cremation is the dominant burial rite in the first two centuries AD.

While this study provides an initial demonstration that the well-established protocol for sampling and cleaning calcined bone for strontium isotope analysis, as outlined by [33], could also be sufficient for the analysis of lead isotope ratios in cremated bone, its novelty necessitates further testing and replication to establish its robustness and reliability. Despite this, it is clear that remains interred in or with a lead/leaded object are significantly contaminated with exogenous lead that cannot be removed during the cleaning process. Therefore, analysis of lead isotope ratios in bone from these types of contexts is not recommended to establish geographical origins of the interred.

## Supplementary Material



## Data Availability

All data is included in the supplementary material [88] and will be deposited in IsoArch (https://isoarch.org/), an open-access resource for archaeological isotope data.
