# Supplementary material for: Decoding the dead: new combined application of strontium and lead isotope analysis to Romano-British cremations from Colchester (UK)
Source: R Soc Open Sci. 2026 Nov 4;13(11):260591. doi: 10.1098/rsos.260591 (PMC13730631; doi:10.1098/rsos.260591)
Supplement: Supplementary file 1 [file rsos.260591.zip › rsos.260591/Table 2.docx]

|  | | **OTIC CAPSULE** | | | | **OUTER PETROUS** | | | | | | | | | | | | | |
| --- | --- | --- | --- | --- | --- | --- | --- | --- | --- | --- | --- | --- | --- | --- | --- | --- | --- | --- | --- |
|  |  |  |  |  |  |  |  |  |  |  |  |  |  |  |  |  |  |  |  |
| **DU**  **Lab #** | **Sample #** | **Sr ppm** | **^87^Sr/^86^Sr** | **2 SE** | **Pb ppm** | **Sr ppm** | **^87^Sr/^86^Sr** | **2 SE** | **Pb ppm** | **^206^Pb/^204^Pb** | **2 SE** | **^207^Pb/^204^Pb** | **2 SE** | **^208^Pb/^204^Pb** | **2 SE** | **^207^Pb/^206^Pb** | **2 SE** | **^208^Pb/^206^Pb** | **2 SE** |
| Col_1 | 1928.363.3 | 112 | 0.7101864 | 0.00001 | 2.2 | 126 | 0.709667 | 0.000013 | 2.4 | 18.36949 | 0.00079 | 15.64247 | 0.00075 | 38.39679 | 0.00253 | 0.85154 | 0.00002 | 2.090221 | 0.00007 |
| Col_2* | 1930.480.1 | 93.0 | 0.7095264 | 0.001246 | 275 | 100 | 0.709583 | 0.000009 | 123 | 18.42803 | 0.00058 | 15.64618 | 0.00050 | 38.45825 | 0.00142 | 0.84903 | 0.00001 | 2.086907 | 0.00003 |
| Col_3* | 1930.492.1 | 74.0 | 0.7102164 | 0.000011 | 2040 | 71 | 0.710398 | 0.000009 | 541 | 18.41314 | 0.00050 | 15.64210 | 0.00053 | 38.42950 | 0.00147 | 0.84951 | 0.00001 | 2.087085 | 0.00003 |
| Col_4 | 2000.72.526.1 | 44.0 | 0.7098164 | 0.000018 | 3.0 | 102 | 0.709761 | 0.000009 | 2.9 | 18.45158 | 0.00082 | 15.64882 | 0.00080 | 38.47609 | 0.00255 | 0.84809 | 0.00001 | 2.085217 | 0.00006 |
| Col_5 | 34.81.1 | 76.1 | 0.7112284 | 0.000014 | 8.3 |  |  |  |  |  |  |  |  |  |  |  |  |  |  |
| Col_6 | Jarmin.1.1 | 122 | 0.7126744 | 0.000014 | 9.6 | 119.9 | 0.710740 | 0.000009 | 7.8 | 18.42150 | 0.00064 | 15.64567 | 0.00059 | 38.45738 | 0.00157 | 0.84932 | 0.00001 | 2.087660 | 0.00004 |
| Col_7 | Jarmin.3.1 | 109 | 0.7096774 | 0.000013 | 9.6 |  |  |  |  |  |  |  |  |  |  |  |  |  |  |
| Col_8 | Jarmin.89.1 | 73.4 | 0.7119194 | 0.00001 | 2.1 |  |  |  |  |  |  |  |  |  |  |  |  |  |  |
| Col_9 | JOS.118.1 | 59.3 | 0.7102724 | 0.000015 | 7.8 | 87.6 | 0.710399 | 0.000009 | 1.6 | 18.43366 | 0.00120 | 15.65174 | 0.00125 | 38.47076 | 0.00401 | 0.84909 | 0.00002 | 2.087055 | 0.00008 |
| Col_10 | JOS.32.1 | 81.2 | 0.7100544 | 0.000013 | 16.9 |  |  |  |  |  |  |  |  |  |  |  |  |  |  |
| Col_11 | JOS.320.1 | 82.6 | 0.7098994 | 0.000013 | 0.8 | 100.1 | 0.710230 | 0.000012 | 0.5 |  |  |  |  |  |  |  |  |  |  |
| Col_12 | JOS.446.1 | 44.8 | 0.7091924 | 0.000009 | 47.1 | 83.4 | 0.709234 | 0.000006 | 34.4 | 18.47239 | 0.00059 | 15.67298 | 0.00055 | 38.63065 | 0.00148 | 0.84846 | 0.00001 | 2.091284 | 0.00003 |
| Col_13 | JOS.485.1 | 79.9 | 0.7102484 | 0.00001 | 0.6 | 84.1 | 0.709792 | 0.000009 | 1.7 | 18.44043 | 0.00052 | 15.64588 | 0.00047 | 38.44320 | 0.00129 | 0.84847 | 0.00001 | 2.084778 | 0.00004 |
| Col_14 | JOS.491.1 | 70.3 | 0.7093834 | 0.000012 | 5.2 |  |  |  |  |  |  |  |  |  |  |  |  |  |  |
| Col_15 | JOS.781.1 | 98.7 | 0.7099904 | 0.00001 | 4.0 | 91.1 | 0.710220 | 0.000009 | 4.3 | 18.49114 | 0.00057 | 15.65829 | 0.00059 | 38.56600 | 0.00163 | 0.84680 | 0.00001 | 2.085621 | 0.00004 |
| Col_16 | PC.611.1 | 83.3 | 0.7098554 | 0.000012 | 0.4 |  |  |  |  |  |  |  |  |  |  |  |  |  |  |
| Col_17 | PC.612.1 | 109 | 0.7085664 | 0.000012 | 5.9 | 186.8 | 0.708475 | 0.00001 | 8.4 | 18.50119 | 0.00066 | 15.65430 | 0.00067 | 38.54410 | 0.00187 | 0.84613 | 0.00001 | 2.083333 | 0.00004 |
| Col_18 | PC.727.1 | 122 | 0.7108424 | 0.000011 | 1.9 | 191.7 | 0.710620 | 0.00001 | 6.2 | 18.47991 | 0.00051 | 15.66861 | 0.00054 | 38.61065 | 0.00159 | 0.84787 | 0.00001 | 2.089341 | 0.00004 |
| Col_19 | PC.752.1 | 77.3 | 0.7095214 | 0.000012 | 0.6 | 108.9 | 0.709555 | 0.000008 | 4.5 | 18.53028 | 0.00058 | 15.66710 | 0.00058 | 38.63693 | 0.00174 | 0.84548 | 0.00001 | 2.085048 | 0.00004 |
| Col_20* | 1913.2728.1.2 | 110 | 0.7112114 | 0.000012 | 3029 |  |  |  |  |  |  |  |  |  |  |  |  |  |  |
| Col_21* | 1923.4606.1 | 77.6 | 0.7094404 | 0.000015 | 59.3 | 96.3 | 0.709722 | 0.00001 | 137 | 18.46784 | 0.00047 | 15.65437 | 0.00050 | 38.51200 | 0.00151 | 0.84767 | 0.00001 | 2.085363 | 0.00004 |
| Col_22* | 2020.16.1 | 71.9 | 0.7104424 | 0.00001 | 987 |  |  |  |  |  |  |  |  |  |  |  |  |  |  |

**Table 2: Strontium and lead isotope and concentration data for the otic capsule and outer surface of the petrous bone.**

* Pewter or lead ossuary/burial container
